# Monitoring serum IL-18 levels is useful for treatment of a patient with systemic juvenile idiopathic arthritis complicated by macrophage activation syndrome

**DOI:** 10.1186/1546-0096-9-15

**Published:** 2011-07-13

**Authors:** Tomonari Shigemura, Takashi Yamazaki, Yosuke Hara, Jing-Ni Ou, Anne M Stevens, Hans D Ochs, Kenichi Koike, Kazunaga Agematsu

**Affiliations:** 1 Department of Pediatrics, Shinshu University School of Medicine, Matsumoto 390-8621, Japan; 2 Seattle Children's Research Institute, 1900 9th Avenue, Seattle, WA 98101, USA; 3 Department of Infection and Host Defense, Shinshu University, Graduate School of Medicine, Matsumoto 390-8621, Japan

## Abstract

Systemic juvenile idiopathic arthritis (sJIA) is a systemic inflammatory disease characterized by arthritis, spiking fever and a skin rash that is frequently complicated by macrophage activation syndrome (MAS), a life-threatening disorder. We report a 22-month-old girl with sJIA who developed severe MAS but was successfully treated with corticosteroids, cyclosporin A, and non-steroidal anti-inflammatory drugs by monitoring serum IL-18 levels. IL-18 is an extremely useful cytokine for monitoring the activity of sJIA and MAS, and serum IL-18 can be used as an indicator for the effectiveness of treatment and the decision to discontinue therapy.

## Background

Systemic juvenile idiopathic arthritis (sJIA) is a disease of unknown etiology characterized by arthritis and systemic symptoms starting before the age of 16. The most characteristic feature at onset is spiking fever, which is often associated with a skin rash [1]. Macrophage activation syndrome (MAS) is a life-threatening complication of chronic rheumatic diseases during childhood that is seen most commonly in sJIA. Symptoms of MAS include fever, hepatosplenomegaly, lymphoadenopathy, profound depression of all 3 blood cell lines, abnormal liver function, intravascular coagulopathy, and central nervous system dysfunction. MAS is thought to be caused by the activation and uncontrolled proliferation of T lymphocytes and macrophages, resulting in an unrestricted release of inflammatory cytokines [2]. Given that MAS can follow a rapidly fatal course, prompt diagnosis by its clinical and laboratory features and immediate therapeutic intervention are critical for survival [2].

IL-18 was originally described as an INF-γ-inducing factor produced mainly by activated macrophage lineage cells [3]. IL-18 stimulates a variety of inflammatory responses, enhances proliferation and activity of T cells and natural killer (NK) cells, and shifts Th-cell balance towards a Th1 response [4]. In combination with IL-12, IL-18 strongly stimulates T lymphocytes, NK cells, and macrophages to produce IFN-γ [5]. Recently, highly elevated serum levels of IL-18 have been reported in patients with sJIA [6,7].

In the present case, we investigated the utility of serum IL-18 as a marker for disease activity in a patient with sJIA complicated by MAS.

## Case report

A 22-month-old girl was transferred to our hospital from a rural hospital with a 2-week history of fever, rash, mild arthritis in bilateral wrist, knee, and ankle joints, and pain involving the entire body despite therapy with intravenous antibiotics and gamma globulin. On admission, her transaminases, LDH, CRP, sIL-2R and ferritin levels were all elevated (AST 139 U/l, ALT 56 U/l, LDH 1,110 U/l, CRP 10.72 mg/dl, sIL-2R 8,440 U/ml and ferritin 10,284 ng/ml). She had thrombocytopenia and coagulation disturbance; her platelet count, fibrinogen levels, and fibrinogen-fibrin degradation product (FDP-DD) levels were 108,000/μl, 211.9 mg/dl, and 24.8 μg/ml (normal <1.0 μg/ml), respectively. Her fever had changed from spiking to a persistent fever 2 days prior to admission to our hospital. Evaluation for bacterial and viral infections, including the Epstein-Barr virus, were negative. Bone marrow aspirates were negative for dysplasia, but were positive for hemophagocytosis.

**Figure 1:**
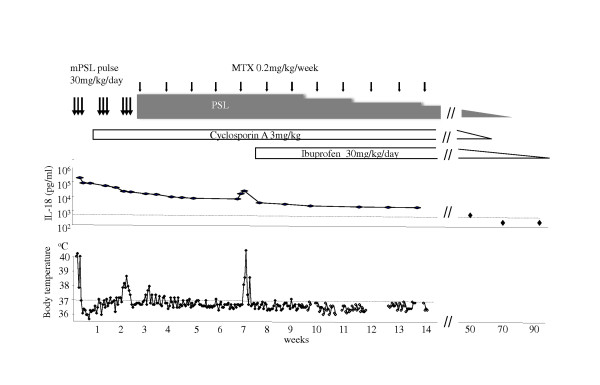
**Treatment course and serum IL-18 levels**. Arrows indicate prednisolone (PSL) pulse therapy and weekly oral administration of methotrexate (MTX)

The patient met the preliminary diagnostic guidelines of MAS [2]. Using her clinical presentation, the 2004 HLH criteria, her serum ferritin level, and her serum sIL-2R level results, we were able to diagnose the child as having sJIA complicated by MAS. Three pulses of methylprednisolone at 30 mg/kg/day and intravenous cyclosporin A at 3 mg/kg/day were initiated (Figure 1). After steroid pulse therapy, prednisone at 0.8 mg/kg/day was commenced. Although the patient's laboratory data improved gradually, her low grade fever persisted for two weeks. Thereafter, methotrexate at 0.4 mg/kg/week was added.

Four weeks after the methylprednisolone pulse therapy and before a dose reduction of the steroids, the girl's fever returned with a mild coagulation disturbance. Her serum FDP-DD level was 13.6 μg/ml. Her transaminases, LDH, CRP, sIL-2R, and ferritin levels were not increased at the time (AST 48 U/l, ALT 22 U/l, LDH 401 U/l, CRP 6.73 mg/dl, sIL-2R 585 U/ml, and ferritin147 ng/ml). On the basis of these clinical and laboratory findings, we made a diagnosis to recurring sJIA without definite MAS. At that time, IL-1 or IL-6 antagonists were not available in Japan. However, as soon as treatment with ibuprofen was initiated without increasing steroid doses, the patient's condition dramatically improved. One and a half years later, all medications were tapered off, and patient has shown no signs of recurrence for two years since cessation of treatment.

## Monitoring serum IL-18

Serum concentrations of IL-18 were determined using a commercially available kit (Human IL-18 enzyme-linked immunoassay kit; BML, Nagoya, Japan)(Figure 1). On admission to our hospital, serum IL-18 was extremely high (284,900 ng/ml, normal range 100-400 ng/ml) and serum IL-6 was elevated at 72.7 pg/ml. Immediately following three courses of steroid pulse therapy, serum IL-18 decreased to 22,064 ng/ml, but remained elevated (over 10,000 ng/ml) from then on. At the time of the fever flare-up, serum IL-18 had increased again to 35,235 ng/ml. After the patient dramatically improved following treatment with ibuprofen, serum IL-18 and IL-6 were 2,000 ng/ml and less than 10 pg/ml, respectively. After that, IL-18 levels gradually improved to normal with serum IL-6 remaining below 10 pg/ml. At that time, we judged we could safely discontinue medication for her sJIA. IL-18 levels of under 400 ng/ml have since persisted for two years without any symptoms.

## Discussion

This report presents a child with sJIA complicated by MAS who was treated successfully while being monitored for serum IL-18 levels at clinically important stages.

Although MAS has been considered a rare complication of sJIA, it is likely more common than previously thought [8-10]. MAS also accounts for a significant proportion of the morbidity and mortality seen in sJIA. Since MAS is a serious condition that can follow a rapidly fatal course, prompt recognition and immediate therapeutic intervention are critical. For the treatment of sJIA complicated by MAS, corticosteroids, cyclosporin A, and non-steroidal anti-inflammatory drugs (NSAIDs) are reported useful. Although methotrexate and tumor necrosis factor antagonists, such as infliximab, are often only partially effective against sJIA, the anti-interleukin 6 receptor antagonist tocilizumab or IL-1 receptor antagonist anakinra have shown good clinical efficacy in small open-label studies of sJIA and adult Still's Disease [11-14]. The use of anakinra to treat the MAS complication of sJIA has been reported [14]. NSAIDs and systemic corticosteroids have historically been the initial mainstays of treatment for sJIA [1] and were administered to our patient. There have not been prior reports of NSAIDs treatment of MAS. Since sJIA did relapse without significant MAS at the time of the fever flare-up, it appeared that ibuprofen was effective for treating sJIA in this case. With the current availability of tocilizumab or anakinra in Japan for sJIA, we employ these drugs as treatment of sJIA recurrence or when disease activity is uncontrollable. We anticipate that these new drugs, tocilizumab and anakinra, may decrease serum IL-18 when decreasing disease activity in sJIA/MAS.

The difficulty in diagnosing sJIA or sJIA complicated by MAS emphasizes the need for more sophisticated diagnostic tools. Given that serum IL-18 is often increased in sJIA and spikes sharply when complicated by MAS, IL-18 may be useful to diagnose MAS following sJIA [6,7]. IL-18 is also helpful in the differentiation of sJIA and Kawasaki disease because of low levels of serum IL-18 in the latter [6]. In contrast, serum IL-6 levels were found to be high both in sJIA and Kawasaki disease, but did not increase further when sJIA patients developed MAS [7]. In our case, we failed to assay serum IL-18 during early state sJIA prior to MAS because serum was not available. However, we were able to confirm the recurrence of sJIA through the elevation of IL-18 levels when her high fever recurred with a minor coagulation disturbance.

The precise criteria for discontinuation of sJIA medication are an important issue for clinicians. Shimizu et al. reported that serum IL-18 levels, but not IL-6, neopterin or CRP, were significantly different in both active and inactive disease states of sJIA without MAS, demonstrating the utility of IL-18 in evaluating disease activity of sJIA [7]. Accordingly, we discontinued medication when IL-18 levels fell to less than 400 ng/ml, and the patient has subsequently been in remission for 2 years to date.

## Conclusion

This case report suggests that evaluating serum IL-18 levels in children with sJIA may be very useful in assessment and treatment of the sJIA as well as with the sJIA complication MAS.

## Competing interests

The authors declare that they have no competing interests.

## Authors' contributions

TS collected and organized data, and drafted the manuscript. TY & YH assisted in data collection and analysis, and contributed to the manuscript. JO & AMS independently reviewed the manuscript. HDO & KK oversaw the project, critically reviewed the manuscript, and provided rheumatologic and immunologic analysis of the data. KA designed the project and drafted the manuscript. All authors read and approved the final manuscript.
